# Therapeutic Hypothermia and Out-of-Hospital Cardiac Arrest in a Child with Hypertrophic Obstructive Cardiomyopathy

**DOI:** 10.1155/2015/796151

**Published:** 2015-03-16

**Authors:** Nancy Spurkeland, Gregory Bennett, Chandran Alexander, Dennis Chang, Gary Ceneviva

**Affiliations:** ^1^Penn State Hershey College of Medicine, Hershey, PA 17033, USA; ^2^Department of Pediatrics, Hershey Medical Center, Hershey, PA 17033, USA; ^3^Department of Pediatrics, Pediatric Cardiology, Hershey Medical Center, Hershey, PA 17033, USA; ^4^Department of Pediatrics, Pediatric Intensive Care, Hershey Medical Center, Hershey, PA 17033, USA

## Abstract

Neurologic outcomes following pediatric cardiac arrest are consistently poor. Early initiation of cardiopulmonary resuscitation has been shown to have positive effects on both survival to hospital discharge, and improved neurological outcomes after cardiac arrest. Additionally, the use of therapeutic hypothermia may improve survival in pediatric cardiac arrest patients admitted to the intensive care unit. We report a child with congenital hypertrophic obstructive cardiomyopathy and an out-of-hospital cardiac arrest, in whom the early initiation of effective prolonged cardiopulmonary resuscitation and subsequent administration of therapeutic hypothermia contributed to a positive outcome with no gross neurologic sequelae. Continuing efforts should be made to promote and employ high-quality cardiopulmonary resuscitation, which likely contributed to the positive outcome of this case. Further research will be necessary to develop and solidify national guidelines for the implementation of therapeutic hypothermia in selected subpopulations of children with OHCA.

## 1. Introduction

Epidemiologic studies regarding the outcome of out-of-hospital cardiac arrest (OHCA) in children show heterogeneous results. OHCA in Netherlands resulted in 24% (12/51) survival to hospital discharge; however, larger studies in the United States, Canada, and Japan revealed overall survival rates ranging from 6.4% (40/621) to 8.5% (700/8240) [1–3]. While recovery rates from OHCA in children are low, they are even lower for those with preexisting cardiac disease [4, 5]. Although bystander cardiopulmonary resuscitation (CPR) has been associated with improved survival following cardiac arrest, prolonged CPR is associated with poor outcomes [2, 6]. Therapeutic hypothermia as a method of neuroprotection following cardiac arrest is gaining popularity within the field of pediatrics [1]. There is paucity of peer-reviewed medical literature detailing successful therapeutic hypothermia utilization in children with preexisting cardiac comorbidities [2].

We describe a 12-year-old female with a known history of hypertrophic obstructive cardiomyopathy (HOCM) who responded to therapeutic hypothermia following prolonged CPR for sudden cardiac arrest.

## 2. Case Report

A 12-year-old Caucasian female with a known history of HOCM (asymmetric, obstructive type) and Wolff-Parkinson-White syndrome (WPW) without supraventricular tachycardia collapsed while waiting at the school bus stop. She was unresponsive and pulseless, raising concern for cardiac arrest. Her father, a trained emergency medical technician (EMT) who witnessed the event, immediately performed CPR in the field for an estimated 30 minutes until emergency medical personnel arrived. Cardiac rhythm analysis revealed ventricular fibrillation. Manual external defibrillation (DC cardioversion) was utilized twice during transport to a local hospital, the second of which returned her to a sinus rhythm. She remained unconscious and was hemodynamically unstable, requiring endotracheal intubation, multiple normal saline intravenous boluses, and pressor therapy. She was stabilized and subsequently transferred to the PICU in our tertiary care children's hospital for further evaluation and treatment.

The patient's EKG upon admission showed a QTc of 508 milliseconds, which was prolonged from her baseline of 458 milliseconds. Due to her witnessed collapse and prompted initiation of CPR, a therapeutic hypothermia protocol was initiated with a 48-hour cooling regimen. Goal temperatures were between 32.5 and 33 degrees Celsius during cooling. The patient was maintained on antiepileptic therapy for seizure prophylaxis, as well as fentanyl and midazolam drips for appropriate sedation while undergoing therapeutic hypothermia. An EEG performed within 24 hours of admission showed “intermittent diffuse background slowing with no epileptiform discharges, focal features, or asymmetries.” The patient was rewarmed at hour 48 at a rate of 1 degree Celsius every six hours over the course of the ensuing 48 hours, ultimately accomplishing euthermia between 36.5 and 37.5 degrees Celsius. Extubation trials failed on hospital day 5 due to delirium and shallow work of breathing; following CPAP trials on hospital day 10, successful extubation occurred with no gross neurologic, renal, or hepatic insults noted on clinical and laboratory evaluation. An MRI (multiplanar, multisequence MRI without contrast) of the brain at day 10 revealed no structural abnormalities or evidence of gross ischemia. An adenosine challenge was also conducted in the electrophysiology laboratory, which revealed that the cardiac preexcitation was secondary to a fasciculoventricular pathway not capable of participating in tachycardia. Therefore, it was determined that this accessory pathway did not contribute to tachycardia leading to cardiac arrest in this instance. An automated implantable cardioverter defibrillator was placed on hospital day 15. The patient was discharged on hospital day 17 with no neurological sequelae to date. Follow-up EKGs have demonstrated a return to her baseline QTc of 460 milliseconds. She has experienced no recurrent episodes of torsades de pointes, ventricular tachycardia, or ventricular fibrillation.

## 3. Discussion

This case correlates with existing studies that have demonstrated a survival benefit of early initiation of CPR [3]. It is theorized that cardiac arrests in children often result in poor outcomes when they occur out-of-hospital due to delays in initiation of CPR, nonavailability of advanced life support capabilities, and a lack of the necessary facilities to implement therapeutic hypothermia [4]. Early effective CPR reduces the time during which patients are hypoxic or anoxic.

Over the past half century, therapeutic hypothermia has been increasingly used as an adjunctive component of resuscitation in a variety of critically ill pediatric populations with mixed results. A multicenter, multinational randomized control trial comparing the efficacy of therapeutic hypothermia to normothermia in pediatric traumatic brain injury (TBI) patients demonstrated no mortality benefit associated with the use of hypothermia [5]. Despite documented relative successes in animal models and neonates, favorable neurologic outcomes occur in as few as one-third of pediatric patients who undergo therapeutic hypothermia after surviving cardiac arrest [6]. Other studies have reported concern regarding the use therapeutic hypothermia in patients with QTc prolongation due to the increased risk of ventricular fibrillation [7]. However, a recent report documented the successful use of therapeutic hypothermia as a means of neuroprotection in a child with OHCA and congenital long QT syndrome [8]. The use of therapeutic hypothermia in our patient who developed prolonged QTc as a result of an OHCA supports its safety in patients who may be predisposed to serious arrhythmias [9].

Much of the current peer-reviewed evidence for the utilization of therapeutic hypothermia in young children is based on patients who suffered cardiac arrest secondary to a respiratory etiology (most commonly drowning or choking) as opposed to those of cardiac origin [4]. Likewise, population based studies regarding pediatric OHCA have not mentioned the specific cardiac causes, nor the adaptation of therapeutic hypothermia in postresuscitation treatment, which further highlights the rarity of our patient's case [3, 10]. In a prospective study describing the epidemiology and outcomes after pediatric out-of-hospital cardiac arrest, only 9% (54/601) of patients suffered cardiac arrest from ventricular fibrillation [6]. To our knowledge, none of these children with documented cardiomyopathy were reported to have HOCM.

This case is an illustration of the utility of both high-quality CPR in the field and therapeutic cooling in the PICU in a child with OHCA and accompanying congenital heart disease. Continuing efforts should be made to promote and employ high-quality CPR in the general population, which likely contributed to the positive outcome in this instance. Further research is necessary to develop guidelines for routine utilization of therapeutic hypothermia in selective groups of children with OHCA like those with underlying cardiac etiology.

## Abbreviations

HOCM: Hypertrophic obstructive cardiomyopathy
OHCA: Out-of-hospital cardiac arrest
TBI: Traumatic brain injury
WPW: Wolff-Parkinson-White syndrome.
